# Assessment of mental and behavioural non-motor symptoms of Parkinson’s Disease using Artificial Intelligence (AI): a systematic review

**DOI:** 10.1038/s43856-025-01304-9

**Published:** 2026-02-09

**Authors:** Shantao Chloe Chou, Cen Cong, Rosiered Brownson-Smith, Madison Milne-Ives, Edward Meinert

**Affiliations:** 1 School of Public Health, Imperial College London, London, UK; 2 Translational and Clinical Research Institute, Newcastle University, Newcastle upon Tyne, UK; 3 Department of Sport, Exercise and Rehabilitation, Faculty of Health and Life Sciences, Northumbria University, Newcastle upon Tyne, UK; 4 Centre for Health Technology, School of Nursing and Midwifery, University of Plymouth, Plymouth, UK; 5 Department of Primary Care and Public Health, School of Public Health, Imperial College London, London, UK; 6 Gnosis Health Limited, The Catalyst, 3 Science Square, Newcastle Helix, Newcastle upon Tyne, UK

**Keywords:** Parkinson's disease, Prognosis, Medical research

## Abstract

**Background:**

Parkinson’s disease is a progressive neurodegenerative disorder with both motor and non-motor symptoms. Mental and behavioural non-motor symptoms such as cognitive impairment, sleep disturbances, depression, and anxiety greatly affect quality of life but remain difficult to assess with traditional tools. Artificial intelligence has shown potential in healthcare, yet its role in evaluating these symptoms in Parkinson’s disease remains under-reviewed. This systematic review aims to evaluate the performance of artificial intelligence tools in diagnosing, assessing, and managing these symptoms.

**Methods:**

Five databases (Medline, Embase, Scopus, Web of Science and PubMed) were searched up to June 2024 for peer-reviewed studies applying artificial intelligence to mental or behavioural symptoms in adults with Parkinson’s disease. Studies published before 2010 or lacking artificial-intelligence technologies were excluded. Study quality and risk of bias were assessed using QUADAS-2. Extracted data include study objectives, data sources, algorithms, best model, and diagnostic performance (accuracy, sensitivity, specificity). The study received no external financial support.

**Results:**

Here we show sixteen studies examine cognitive impairment and seven examine sleep disorders. However, only three studies focus on depression and one on anxiety, revealing a research gap. No meta-analysis was performed due to heterogeneity.

**Conclusions:**

Artificial intelligence shows promise for assessing mental and behavioural symptoms in Parkinson’s disease, particularly cognitive and sleep disorders. Multimodal models demonstrate higher accuracy than single-source models, though external validation is necessary. The limited studies on depression and anxiety reflect existing diagnostic challenges and data limitations. Future research should refine diagnostic tools and expand multimodal approaches to these symptoms.

## Plain Language Summary

Parkinson’s disease causes both movement problems and non-movement symptoms such as difficulties with thinking, decision-making, memory, sleep, depression, and anxiety. These symptoms are common and deeply affect quality of life for people living with Parkinson’s, but they are often missed or underestimated by traditional clinical assessments. We systematically examined whether artificial intelligence could help detect and monitor these symptoms.We analysed twenty-seven studies and found that artificial intelligence shows promise for identifying thinking and sleep-related problems, but very few studies examined depression or anxiety. The findings suggest that combining different types of patient data improves accuracy, but more validation is needed before these tools can be used in clinical settings to help doctors provide better care.

## Abstract (web summary)

Chou et al. systematically review 27 studies using artificial intelligence to assess mental and behavioural non-motor symptoms in Parkinson’s disease. Multimodal models outperform single-source approaches but require external validation, while limited research on depression and anxiety reflects a broader research gap in the field.

## Introduction

Parkinson’s disease (PD) is a progressive neurodegenerative disorder characterised by both motor symptoms and non-motor symptoms (NMS)^1^ While motor symptoms, such as tremors, rigidity, impaired balance, freezing of gait, and hypokinetic dysarthria remain the primary focus of PD diagnosis and management, NMS, such as cognitive impairment, sleep disorders, anxiety, and depression can significantly affect patient well-being. Yet, these are often overlooked^2^ In 2019, over 8.5 million people were diagnosed with PD globally^3^, with studies reporting that up to 75% of PD patients experience sleep disorders, 50% experience depression, 40% experience anxiety, and 20–50% have cognitive impairment^4^ These symptoms not only contribute to disease burden but also reduce quality of life and complicate care delivery.

Moreover, the global burden of PD has risen sharply, with 5.8 million disability-adjusted life years (DALYs) and 329,000 deaths in 2019, reflecting an over 100% increase since 2000^5^ PD also carries a significant economic burden, with annual costs of $52 billion in the US and £20,123 per person in the UK, including direct and indirect costs^6,7^ Due to PD’s progressive nature^1^, the symptom severity may worsen over time, making treatment more challenging. As a result, PD increasingly challenges healthcare systems and caregivers, requiring significant resources for disease management.

Current assessment of NMS relies heavily on clinical interviews, standardised questionnaires, and medical tests. For example, depression is commonly diagnosed using the Hamilton Depression Rating Scale (HDRS), while anxiety is evaluated with the Hamilton Anxiety Rating Scale (HAM-A) or the Hospital Anxiety and Depression Scale (HADS-A). Cognitive impairment is typically assessed with the Montreal Cognitive Assessment (MoCA), and sleep disturbances are diagnosed through polysomnography (PSG). However, these tools were not designed specifically for PD, which may limit their relevance. For instance, some mental health tools fail to distinguish PD-related mental health symptoms from primary psychiatric disorders^8,9^ Additionally, cognitive tests, such as MoCA can be biased by education levels even after variable adjustments, and the standard 1-point correction may not be sufficient across different settings^10^ Although PSG is accurate, it is costly and resource-intensive, with inconsistencies arising from subjective reporting and clinician interpretation.

To address these challenges, Parkinson-specific instruments, such as the Parkinson Anxiety Scale (PAS), the PD Cognitive Rating Scale (PD-CRS), and the Movement Disorder Society-Unified PD Rating Scale (MDS-UPDRS) have been developed to better assess relevant PD symptoms. Nevertheless, previously described challenges remain. Therefore, AI has been further explored as a complement to current assessments, to make better use of the data they generate, and potentially improve diagnostic accuracy.

AI has shown promise in PD research for its ability to process large datasets and identify patterns. While much of the existing work has focused on motor symptoms, researchers have also begun investigating its application in NMS. For example, a systematic review by Sun et al.^11^ examined how machine learning models combining clinical and magnetic resonance imaging (MRI) data could detect cognitive impairment in PD. However, such efforts have typically focused on individual symptoms. To our knowledge, no comprehensive review has systematically evaluated how AI has been applied to mental and behavioural NMS as a group, particularly cognitive impairment, anxiety, depression, and sleep disorders, despite their substantial prevalence and clinical impact.

This systematic review, therefore, aims to systematically evaluate how AI tools have been applied to diagnose, classify, or predict these four key NMS in PD. We focus on summarising the reported performance of these tools, including their accuracy, sensitivity, and specificity, concerning outcomes, such as symptom classification, risk prediction, and disease diagnosis.

In this systematic review of 27 studies, we find that artificial intelligence shows promising performance for identifying cognitive impairment and sleep disorders in PD, especially when multimodal approaches are used. However, the application of AI to depression and anxiety in PD remains limited, highlighting a research gap. While multimodal models combining different data types demonstrate superior performance compared to single-modality approaches, the contribution of individual features varies across contexts, emphasising the importance of optimising feature selection during model development. Nevertheless, variability in model performance between training and testing sets raises concerns about overfitting and limited generalisability. These findings suggest that AI tools have the potential to improve assessment of NMSs in PD, but further validation in real-world settings is essential before clinical implementation.

## Methods

### Search strategy

A literature search was conducted across five electronic databases up to June 2024: Medline, Embase, Scopus, Web of Science and PubMed. Additional systematic review searches were performed in the PROSPERO and Cochrane Library databases. The search strategy was developed in consultation with a research librarian and incorporated relevant keywords and MeSH terms related to PD, AI, and mental and behavioural NMS (Supplementary Data 1). The strategy was guided by the PICO framework (Table 1) and applied database-specific filters, yielding 5222 articles (Supplementary Data 2).

**Table 1:** PICO table

PICO Component	Description
**Population (P)**	Adult patients with PD who experience non-motor mental and behavioural symptoms, with no specification on gender and ethnicity.
**Intervention (I)**	Any AI technologies that are being utilised to manage mental and behavioural NMSs in PD patients.
**Comparison (C)**	N/A
**Outcomes (O)**	The primary outcomes of interest include the diagnostic accuracy performance metrics of AI tools, which are accuracy, sensitivity, specificity.

### Inclusion and exclusion criteria

This systematic review included peer-reviewed studies from 2010 onward that applied AI to diagnosing and managing mental and behavioural NMSs in adult PD patients. Key exclusion criteria included non-PD conditions, non-AI interventions, pre-2010 studies, and non-English publications. The full breakdown of inclusion and exclusion criteria is listed in Supplementary Data 3.

### Screening process

**Fig. 1:**
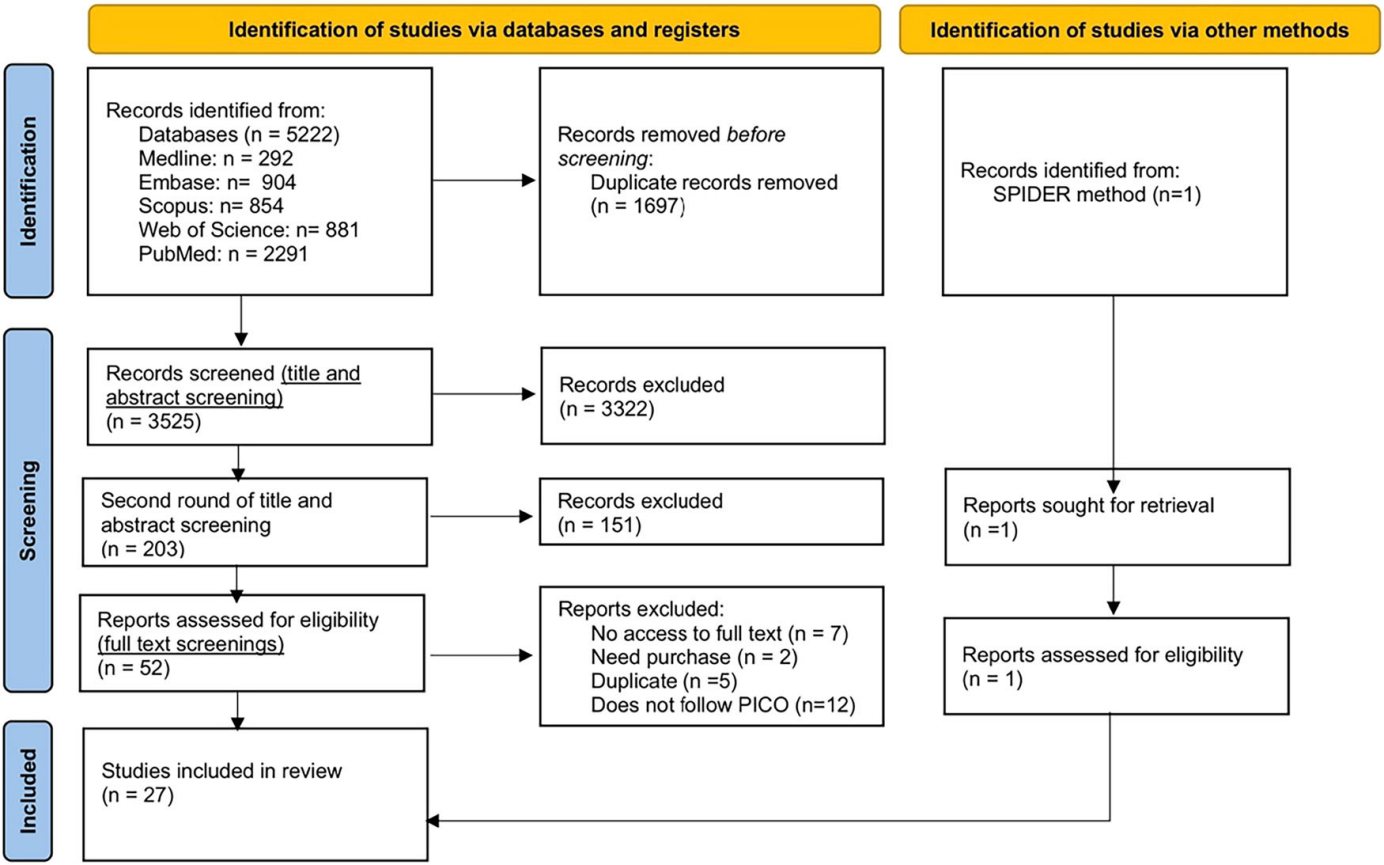
PRISMA flowchart - The search identified 5222 records from five databases and one additional record through the SPIDER method, 27 studies were included in the final review.

A three-stage screening process was conducted based on the protocol by Bounsall et al.^12^, with modifications made to the original protocol (Table 2), and the PRISMA guidelines (Fig. 1). Electronic screening was first applied to filter articles based on the presence of keywords across different fields (any field, abstract, and title), as detailed in Supplementary Data 4, followed by title and abstract, and full-text screening. Reasons for exclusion at the full-text stage are summarised in Supplementary Data 5. All screening steps were performed independently by one reviewer (SC).

**Table 2:** Modifications to the original protocol

Original Protocol	Modified Protocol
- Effectiveness, accuracy, specificity, or sensitivity of method (if reported) - Test training sets of data and their percentages - Effectiveness, efficacy, usability, acceptability, and feasibility outcome measures (if reported)	Focus on accuracy, sensitivity, specificity
Comprehensive review of the range of PD symptoms	Limited to mental and behavioural NMS in PD
PubMed, IEEE Xplore, Institute for Scientific Information’s Web of Science, the Cochrane Library, and Scopus	Embase, Medline, PubMed, Web of Science, and Scopus

### Quality assessment

The Quality Assessment of Diagnostic Accuracy Studies 2 (QUADAS-2) tool was used to evaluate the quality of included studies^13^ QUADAS-2 assesses the risk of bias across four domains: patient selection, index test, reference standard, and flow and timing. Applicability concerns were evaluated for the first three domains (Supplementary Data 6). Each study was rated as “low,” “high,” or “unclear” risk based on predefined criteria (Supplementary Data 7). High-risk ratings suggested significant biases, low-risk ratings suggested minimal concerns, and unclear ratings reflected insufficient information.

### Data Extraction

Data extraction was conducted by one reviewer, SC, using a structured table (Table 3). The table was developed based on the research objectives and the PICOS framework, capturing study objectives and reported performance metrics.

**Table 3:** Data Extraction Framework for Included Studies

Category	Data to be extracted
**General study information**	Title
	Authors
	Year of publication
**Data collection & processing**	Data sources
	Algorithm
**Results**	Accuracy (%)
	Sensitivity (%)
	Specificity (%)
	Best model
**Notes**	Notes

The following performance metrics were used to evaluate the AI models: accuracy: the proportion of correct predictions (both true positives and true negatives) among all predictions Sensitivity: the proportion of actual positive cases correctly identified (true positive rate) Specificity: The proportion of actual negative cases correctly identified (true negative rate)

### Data analysis and synthesis

Due to heterogeneity in outcome measures and methodologies, a meta-analysis was not feasible. Instead, a descriptive analysis summarised study characteristics, methodologies, and outcomes across the four symptom categories (cognitive impairment, depression, sleep disorders, and anxiety). Supplementary Data 8 presents the extraction of the included studies, documenting their objectives, data sources, algorithms, performance metrics, and best performing model. This allows the synthesis of observed patterns in algorithm performance, providing a comprehensive overview of the current literature on AI’s diagnostic performance in analysing mental and behavioural NMS in PD. Table 4 summarises the best-performing model from each study. Models were excluded if multiple models performed equally well, or if the best-performing model varied across different tasks within the same study. This systematic review received no external financial support and was not registered.

**Table 4:** Best-performing AI models in included studies

AI Model	Number of Studies as Best Performer	Studies
Support Vector Machine (SVM)	9	Zhang et al. (2021a), Booth et al. (2022), Chen et al. (2022), Yang et al. (2022), Raschella et al. (2023), Beheshti and Ko (2024), Jia et al. (2024), Rechichi et al. (2024), Shu et al. (2024)
Random Forest (RF)	5	Bisgin et al. (2020), Byeon (2020), Chong-Wen et al. (2022), Jeon et al. (2022), Shibata et al. (2022)
LEAPD	2	Espinoza et al. (2022), Anjum et al. (2024)
Artificial Neural Network (ANN)	1	Sorensen et al. (2011)
Correlation-based Feature Subset selection Naïve Bayes (CFS-NB)	1	Morales et al. (2013)
Extra Trees Classifier with ANOVA	1	Hosseinzadeh et al. (2023)
Modified Cole-Kripke Algorithm	1	Ko et al. (2022)
XGBoost	1	Chen et al. (2023)
VMD-Based Convolutional Neural Network (VMD-CNN)	1	Parajuli et al. (2023)
Logistic Regression	1	Jian et al. (2024)
Conditional Inference Forest	1	Harvey et al. (2022)

### SPIDER technique

The SPIDER technique was used to review reference lists from included studies to retrieve more relevant studies. This approach identified one additional article^14^ on cognitive impairment. However, no further studies focusing on AI applications for depression or anxiety in PD were identified through this approach.

### Statistics and reproducibility

Statistical meta-analysis was not performed due to heterogeneity in study methodologies and outcome measures. Instead, a descriptive synthesis was used to identify trends in accuracy, sensitivity, and specificity across studies. To ensure reproducibility, the search strategy, PICO framework, inclusion and exclusion criteria, and data extraction framework were predefined, with details provided in the Supplementary Information. Quality assessment was performed using the QUADAS-2 tool to evaluate risk of bias and applicability concerns across domains.

### Ethics approval and consent to participate

No ethical approval was required for this study as it did not involve human or animal subjects. All sources of data were publicly available.

## Results

### Study Selection

In total, 5222 articles were retrieved from five electronic databases, with one additional record identified using the SPIDER method. After duplicate removal and multi-stage screening (Fig. 1), 27 studies met the inclusion criteria and were included in the final review. During full-text screening, 26 articles were excluded for reasons including lack of access (*n* = 7), need for purchase (*n* = 2), duplication (*n* = 5), and not following PICO criteria (*n* = 12) (Supplementary Data 5).

### Included Studies and Study characteristics

The characteristics of the 27 studies are summarised in Supplementary Data 8, which outlines each study’s objectives, data sources, AI algorithms used, reported accuracy, sensitivity, specificity, best-performing model, and relevant notes. Sixteen focused on cognitive impairment^14–29^, involving 2241 participants; seven on sleep disorders with a total of 687 participants^30–36^; three on depression^37–39^ with 265 participants, and one on anxiety^40^ with 219 participants.

### Data extraction table

Table 4 summarises the best-performing AI model, defined as those achieving the highest accuracy compared to other models tested within the same study. Support Vector Machine (SVM) were the most frequently reported best performer, identified in nine studies, followed by Random Forest (RF) in five studies. However, the overall heterogeneity in best-performing models suggests that the best AI approach depends on the NMS analysed, available data, and patient characteristics.

Supplementary Data 9 summarises the input data sources used across the included studies, categorised into four main categories: neuroimaging, electrophysiological, wearable and portable devices, and clinical assessments or biomarkers. The table allows readers to identify which studies used single-modality input and which combined different types of data, showing the diversity of input data and the use of multimodal approaches in AI model development for PD-related NMS.

The included studies employed various algorithms, with some studies comparing multiple approaches. SVM and RF were most prevalent; SVM was used in 15/27 studies (55.6%), followed by RF in 11/27 studies (40.7%), while newer approaches like deep learning were less commonly used. The full range of algorithms employed is provided in Supplementary Data 10.

The following subsections present results categorised by conditions, summarising the AI methods and their reported accuracy, sensitivity, and specificity. A detailed overview of each study’s objective, input data, algorithms used, and performance metrics is presented in Supplementary Data 8, which can be used to contextualise the findings discussed below.

### Cognitive Impairment

16 out of 27 studies (59.3%) focused on cognitive impairment; these studies varied in study objectives, data sources, and AI models, leading to heterogeneity in reported performance metrics. Among these, eight studies focused on binary classification between PD patients with and without cognitive impairment, with classification accuracy ranging from 71.9 to 100%, sensitivity from 50 to 99.2%, and specificity from 81 to 100%. Five studies predicted future cognitive decline, with accuracy ranging from 74 to 86.7%, sensitivity from 67.7 to 91%, and specificity from 70 to 96.1%, though they varied in their follow-up periods (ranging from 4 to 8 years). The remaining three studies had different objectives: one differentiated between multiple cognitive states (PD-Dementia (PDD), PD-Mild Cognitive Impairment (PDMCI), PD-Cognitive Impairment (PDCI)). Another aimed to predict MoCA scores at year 4 and achieved 83% accuracy. The third study distinguished PD-MCI patients from healthy controls, with no other groups included, and achieved 83.3% accuracy. Several studies^17,20,24,28^ showed that multimodal approaches combining different data types outperformed models using only one data type. This trend was also observed in studies combining multiple features within the same data type, such as combining intravoxel and intervoxel diffusion metrics in Diffusion Tensor Imaging (DTI)^22^ or combining graph frequency features in functional near-infra-red spectroscopy (fNIRS)^29^ Furthermore, RF achieved the highest accuracy in two studies that directly compared multiple machine learning algorithms: one classified PD with Normal Cognition (PDNC) vs. PD-MCI and PDD, and the other classified PDNC vs. PD-MCI among non-demented patients. However, some studies have shown a drop in performance from training to testing sets^18,25,26^, raising concerns about potential overfitting.

### Sleep disorders

Seven studies (25.9%) focused on sleep disorders in PD. For PSG-based studies, Sorensen et al.^30^ achieved 89.8% sensitivity in sleep arousal detection using Artificial Neural Networks (ANN). Bisgin et al.^31^ reported 87.84% accuracy in Rapid Eye Movement (REM) sleep classification using RF combined with feature selection, showing particular strength in REM sleep stages detection. As REM sleep disturbances are considered crucial early evidence of neurodegeneration and may support the diagnosis of PD^31^, PSG-based AI models targeting sleep patterns may provide valuable insights for early PD detection.

Two studies focused on Rapid Eye Movement Sleep Behaviour Disorder (RBD) prediction, and both demonstrated the superiority of RF over other algorithms despite having different study objectives. Byeon’s^32^ model achieved 71% accuracy, 79% sensitivity, and 67% specificity in identifying high-risk RBD-PD patients. Chong-wen’s^34^ RF model demonstrated higher accuracy (83.05%) and specificity (93.06%) but lower sensitivity (67.39%), which was 100% in the training set. Both studies identified predictive factors for RBD in PD patients with significant overlap, such as age, cognitive function, and motor scores. As RBD can precede PD for several years and is associated with more aggressive PD phenotypes^35^, AI-based models for RBD detection and prediction may offer valuable opportunities for earlier intervention and more accurate risk prediction.

Studies using wearable devices also showed promising results: Ko et al.^33^ achieved 84.5% accuracy in awake/sleep detection using smartwatch sensors, Raschella et al.^35^ reported 96.2–100% accuracy in RBD classification using wrist actigraphy, and Rechichi et al.^36^ achieved 96.2% accuracy for PD detection and 85.7% for sleep quality classification using Inertial Measurement Units (IMU). These wearables are valuable for continuously tracking PD related sleep disorders.

### Depression

Three studies (11.1%) focused on differentiating depression in PD (DPD) from non-depressed PD (NDPD), healthy control (HC), and non-PD depression. Zhang et al.^37^ showed high accuracy across different classification tasks: DPD vs HC at 100% (SVM), NDPD vs HC at 96% (Lasso), and DPD vs NDPD at 90% (RF). With Linear predictive coding of EEG Algorithm for PD (LEAPD), Espinoza et al.^38^ reported 97% accuracy in distinguishing DPD from NDPD and 100% accuracy for DPD vs non-PD depression. Using SVM, Yang et al.^39^ reported 73% accuracy, 88% sensitivity, and 57% specificity for differentiating DPD from NDPD in the test set, with a significant drop in specificity compared to the training set (from 73- to 57%).

### Anxiety

Only one study^40^ focused on anxiety in PD (3.7%). Combining clinical and structural MRI features, the study reported 88.0% accuracy, 86.0% precision, and 81.0% sensitivity in identifying anxiety in PD using a SVM. This multimodal model showed improved accuracy compared to models using either data type alone, highlighting its importance.

### Overall findings

Overall, this systematic review shows that cognitive impairment in PD has received the most attention in AI-based studies, followed by sleep disorders. In contrast, the application of AI to depression and anxiety remains relatively under-analysed, suggesting a gap in current research.

Furthermore, RF and SVM are commonly used across studies. RF was evaluated in 11/27 studies (40.7%) with multiple-algorithm comparisons, ranking as the best-performing model in 5 studies based on their specific objectives. This suggests RF may be well-suited to analysing the complex nature of mental and behavioural NMS in PD. SVM appeared in 15/27 studies (55.6%), achieving the best performance in 9 studies according to their specific objectives. However, 7 of these 9 studies used SVM as the only model, introducing potential limitations for comparison with other models.

## Discussion

This systematic review of 27 studies examined AI applications across four major NMSs in PD: cognitive impairment (59.3%), sleep disorders (25.9%), depression (11.1%), and anxiety (3.7%). Cognitive impairment and sleep disorders in PD demonstrated more extensive AI applications, characterised by comparisons of multiple algorithms and promising results. Evaluations of AI with cognitive applications have evolved from simple algorithm comparisons to multimodal approaches over the years. Similarly, sleep disorder research shows an evolution from complex PSG data analysis to more accessible wearable devices that can offer continuous monitoring. This trend is supported by Iakovakis et al.^41^, who showed that sleep data from smartwatches in real-world settings could accurately distinguish early PD, highlighting the potential of wearable devices. Prashanth et al.^42^ also showed that self-reported olfactory loss and RBD questionnaires could successfully distinguish early PD, demonstrating the value of accessible, non-invasive data sources for AI models. In contrast, AI applications for depression and anxiety in PD (though demonstrating positive results) are in earlier stages, with most studies (2/3 for depression, 1/1 for anxiety) evaluating a single algorithm for a given objective rather than systematically comparing multiple models. The limited research on anxiety in PD highlights this as an under-reviewed area needing more investigation.

A meta-analysis was not performed in this systematic review due to substantial heterogeneity identified across studies. Supplementary Data 8 presents the data necessary for a descriptive analysis of AI model performance by summarising reported metrics for each study. Across studies, several key trends that have significant implications for future research were observed. Five studies^17,20,24,28,40^ in this systematic review showed that multimodal models combining different data types demonstrated better performance compared to single-modality models. Improvements were reported across various metrics: accuracy, sensitivity, and specificity^17,20^; accuracy alone^24,40^; and AUC and specificity^28^ Several studies in this systeamtic review demonstrated that the contribution of different input features to predictive performance can vary, highlighting the need for careful feature selection and evaluation during model development. For example, Hosseinzadeh et al.^24^ found that clinical features were the dominant predictors, with imaging features providing only marginal improvement in accuracy, whereas Huang et al.^27^ showed that certain neuroimaging features alone could achieve performance comparable to models combining both clinical and imaging data. Similarly, Jeon et al.^14^ found that combining MoCA domain scores with cognitive complaints outperformed the traditional MoCA cutoff, but adding depression scores did not improve accuracy, suggesting that not all variables contribute equally to model performance. In another example, Chen et al.^19^ reported that dimensionality reduction using Principal Component Analysis (PCA) improved model accuracy from 84.6 to 100%. Collectively, these findings align with Altham et al.’s^43^ finding on the importance of optimising feature selection. Another observation was the variability in model performance between training and testing sets in some studies^18,25,26,39^, raising concerns about potential overfitting and limited generalisability of certain models. This highlights the need for further validation before clinical applications.

Previous systematic reviews of AI applications in PD have typically focused on individual symptoms. In contrast, this systematic review synthesises research across multiple prevalent NMS, including cognitive impairment, sleep disorders, depression, and anxiety. Our findings address a critical research gap identified by Sun et al.^11^, who observed that almost no qualified studies had integrated radiomics and other biomarkers into machine learning models for cognitive impairment diagnosis in PD. Several recent studies in our systematic review, such as those by Hosseinzadeh et al.^24^ and Jian et al.^28^, successfully demonstrated the superior performance of multimodal models that integrate radiomics features with clinical features. Our findings align with Altham et al.‘s^43^ conclusions that RF algorithms effectively handle complex data, as evidenced by their strong performance across multiple studies^14,21,31,32,34^

Strengths of this systematic review included the comprehensive search strategy across five major databases (Embase, Medline, PubMed, Web of Science, Scopus), the use of the population, intervention, comparison, outcomes (PICO) and PRISMA (Preferred Reporting Items for Systematic Reviews and Meta-Analyses) frameworks to structure the review, and the identification of additional references via spidering. These strategies reduced the risk of missing potentially relevant papers in the initial search. Inclusion and exclusion criteria were clearly defined before commencing screening to increase consistency, and a systematic multi-stage screening process was conducted to focus the review on the most relevant studies to address the research question.

However, several limitations should be noted. Restriction to studies published in English could introduce language bias, but was necessary given available resources. Excluding grey literature has impacted the findings; given the rapid advancement of AI in healthcare, some of the most recent and innovative work might first appear in non-peer-reviewed sources. This is particularly relevant for AI applications for anxiety and depression in PD, which is in earlier stages, as the limited number of studies could have led to an underestimation of current research. This exclusion criteria was implemented to ensure the systematic review focused on high-quality evaluations.

Conducting a meta-analysis would have strengthened our ability to quantify AI performance across studies, but was not possible due to the heterogeneity of models and outcomes. Future systematic reviews should consider conducting a meta-analysis, which would benefit from a less heterogeneous set of included studies, particularly in terms of comparable populations, study objectives, outcomes, and index tests or models. Another limitation is that the systematic review was conducted by a single reviewer, which may have increased the risk of bias and human error in quality assessment. Finally, publication bias may have influenced the findings, as studies with positive results are more likely to be published, potentially leading to an overestimated assessment of AI’s role in managing NMS in PD.

Multimodal models repeatedly demonstrated higher accuracy than models using single data sources for identifying NMS in PD, which indicates that investing in developing these models could help improve PD care. This aligns with a previous study done by Prashanth et al.^44^, who showed that while prior studies had used non-motor, CSF, or imaging markers individually, their study was among the first to combine all three, and resulted in enhanced accuracy in classifying early PD from HC. However, to ensure reliability in real-world settings, external validation of AI tools in PD diagnosis is necessary to support clinical implementation, as several studies observed performance drops in testing sets. Future studies should emphasise rigorous validation using independent datasets and longitudinal studies to capture symptom progression. Given the evidence that NMS are interconnected and often co-occurring. For example, Pearson et al. showed that sleep disorders can worsen cognitive impairment and increase depression risk^45^ Therefore, future research should also focus on early symptom prediction using baseline data and explore AI models that can assess multiple NMS.

The limited number of AI studies identified that focused on depression (*n* = 3) and anxiety (*n* = 1) in PD may be due to broader research gap in the field. For example, Yang et al.^39^ found that over 60% of self-reported depression cases in PD went unrecognised by neurologists using standard tools like the UPDRS. This could result in insufficient reliable training data for AI development, contributing to the low number of published AI studies addressing these symptoms. Similarly, Jia et al.^40^ reported in 2024 that no previous studies had used machine learning to integrate clinical and neuroimaging data for identifying PD-related anxiety. Together, these findings indicate an imbalance in the current literature. As such, future research should develop more accurate diagnostic tools for depression and anxiety in PD, and extend promising multimodal AI approaches from cognitive and sleep disorders to these symptoms. Furthermore, research should expand beyond English-language studies and grey literature to identify additional relevant studies. These approaches will help develop more reliable and clinically useful AI tools for managing NMS in PD patients, ultimately supporting better clinical decision-making and patient outcomes.

This systematic review examined 27 studies assessing the ability of AI tools to diagnose, assess, and support mental and behavioural NMS in PD patients. The available research focuses primarily on cognitive impairment and sleep, while NMS, such as depression and anxiety, are under-explored despite their high prevalence in PD patients. SVM and RF were the most commonly used algorithms and showed promising results across different NMS, though SVM was the only model being tested in many studies. We found evidence of the superior performance of multimodal models that combine different data types compared to single modality models, although the contribution of features varied by context. This indicates that while integrating multiple data sources can enhance diagnostic accuracy, feature selection needs careful planning based on the context. This systematic review suggests potential for AI applications in PD care. The included studies show that AI tools have achieved promising diagnostic performance in research settings for assessing NMS. This is valuable given that traditional diagnostic methods often overlook these symptoms^3,39^ However, to ensure this potential can be realised, robust external validation of models before clinical implementation must be prioritised, as several studies observed performance drops between training and testing sets. Given the interconnected nature of NMS in PD, there is a significant potential benefit for comprehensive patient care solutions in PD from developing AI models capable of simultaneously assessing multiple symptoms. By doing these, future research could improve how NMS are identified and managed in PD patients, leading to more comprehensive and effective patient care.

## Supplementary information

Description of Additional Supplementary files

Supplementary Data 1

Supplementary Data 2

Supplementary Data 3

Supplementary Data 4

Supplementary Data 5

Supplementary Data 6

Supplementary Data 7

Supplementary Data 8

Supplementary Data 9

Supplementary Data 10

## Data Availability

All data supporting the findings of this study are available within the article and its Supplementary Information. The detailed extraction table for all included studies is provided as Supplementary Data 8. Further inquiries can be directed to the corresponding author.
