# Supplementary material for: Assessment of mental and behavioural non-motor symptoms of Parkinson’s Disease using Artificial Intelligence (AI): a systematic review
Source: Commun Med (Lond). 2026 Feb 9;6:101. doi: 10.1038/s43856-025-01304-9 (PMC12894826; doi:10.1038/s43856-025-01304-9)
Supplement: Supplementary file 1 — Description of Additional Supplementary files [file 43856_2025_1304_MOESM1_ESM.docx]

**Description of Additional Supplementary Files**

Supplementary Data 1 – Search terms

Supplementary Data 2 – Search results by database

Supplementary Data 3 – Inclusion and Exclusion Criteria

Supplementary Data 4 – Electronic screening

Supplementary Data 5 – Exclusion counts

Supplementary Data 6 – QUADAS-2 quality assessment

Supplementary Data 7 – QUADAS-2 predefined criteria

Supplementary Data 8 – Data extraction table

Supplementary Data 9 – Data sources for included studies

Supplementary Data 10 – Artificial intelligence models used
